# Effect of Time (Within and Between Days), and Dairy Production Factors on the Impedance Value at 24 Acupuncture Points in Dairy Cows 

**DOI:** 10.3390/ani2030415

**Published:** 2012-08-30

**Authors:** Roel H. Bosma, Shirley C.G. Kalkers-van de Ven, Mauk M. J. den Boer

**Affiliations:** 1 Department of Animal Science, Wageningen University, 6700 AH, Wageningen, The Netherlands; 2 Animal Production Systems Group, Wageningen University, 6700 AH, Wageningen, The Netherlands; E-Mail: shirleyvdven@yahoo.ca; 3 i-HEALTH bv, Apeldoornseweg 47, 6814 BJ Arnhem, The Netherlands; E-Mail: mdb@i-health.com

**Keywords:** dairy, electro-acupuncture, acupuncture points, diagnostics, diurnal, lactation

## Abstract

**Simple Summary:**

Whether electro-acupuncture can contribute to reduced antibiotic use in dairy farming depends, among other things, on effects of time and production factors on the impedance values (IVs) at acupuncture points (APs). We measured the IV at 24 APs located left (L) and right (R) of the bladder (BL) and stomach (ST) meridians. The effect of time of measurement (assessed on six cows in one herd) was confirmed for seven APs, and of production factors (analyzed using 108 cows in three herds) for seven APs. We recommend BL19R, BL20R and BL46-02L as reference values, and BL14L, BL16L and BL17L for diagnostics, and ten other APs for further study.

**Abstract:**

This study evaluated the effect of hour and day of measurement, and of production factors on the impedance values (IVs) at 24 acupuncture points (APs). This is a first step in assessing whether electro-acupuncture can contribute to reduced antibiotic use in dairy farming. The APs studied were left (L) and right (R) points of the bladder (BL) and stomach (ST) meridians. The effect of time was measured in a 3x3 Latin square on six cows in one herd. The effect of production factors was analyzed using 108 cows from three herds for two months. The effect of time excludes BL 14R, 16R, 21R, 22R, 30R, 46-02R, 43-01L and 30L, and ST18 bilaterally for diagnostic use. The contribution of parity, age or lactation period to monthly models of BL21R, 18R and 15R, and ST18R exclude these for diagnostic use. Of the remaining APs, BL19R, BL20R and BL46-02L showed stable IVs and are recommended for reference measurements. APs BL14L, BL16L and BL17L are recommended for diagnostics, and BL 16R, 17R, 18R, 23R, 30R, 15L, 20L, 22L and 29L need further study. Factors contributing to the variation in the IV of several APs were: milk robot, number of inseminations, body condition score, days of the preceding lactation, kg milk and kg milk fat of current and preceding month and preceding year, and milk cell count and urea content.

## 1. Introduction

A pilot study [1] showed that the impedance values (IVs or electrical resistance) at six measured acupuncture points (APs) were lower in dairy cattle herds classified as healthy and robust than in herds with chronic health problems. The relationship was confirmed by the negative correlation between the IVs at five out of the six APs (BL15, BL49 and BL52, bilaterally), the innate immune response, and body condition score. The innate immune response was quantified *in vitro* on a liposaccharide (LPS) medium [2]; body condition was scored between 1 and 5 with a precision of 0.25 by observing body condition at six locations on the Holstein dairy cow in accordance with the standards used on Dutch dairy farms (ribs; spines of backbone; transversal processes of the lumbar vertebrae; the hip; the thurl depression between the hip and pinbones; anal area between tail and pinbones).

Voll [3] pioneered electro-acupuncture (EAP) diagnostics in human complementary medicine. APs are found on meridians (e.g., great governor, bladder) and at paravertebral association (“Shu”) points according to the empirical relationships with organs. Soh [4] published scientific evidence of the meridians and the APs, and of their electrophysiological character. The most distal APs on each of the 12 main meridians are called Ting points. APs coincide with loci of softer skin where vessels and nerves cross the dense connective tissue; this allows localization of the APs. The Soviet aerospace program collected large databases of IVs on 24 Ting points located at the tips of fingers and toes. Abnormal IVs allowed detection of imbalances before clinical symptoms of a disease occurred [5]. Relatively high IVs indicate chronic infections, allergies, poisoning, intoxication from medication, mineral deficiency, vitamin deficiency, enzyme deficiency, and circulation problems. Relatively low IVs indicate local disorders, acute infections, acidosis and/or stress.

A major challenge in dairy farming is reducing the use of antibiotics. Early diagnosis followed by treatment using EAP might help achieve this. Before EAP can be used for diagnosis and treatment, it is necessary to identify the normal IVs of the APs and also the APs whose IVs vary only slightly in response to physiological conditions, such as stage of lactation, and time effects, such as the diurnal rhythm. We therefore designed two experiments: the first was to ascertain whether or not the IVs at 24 selected APs vary diurnally, and the second was to identify the normal IVs and the APs that vary in response to farm and physiological conditions. 

## 2. Experimental Section

**Figure 1:**
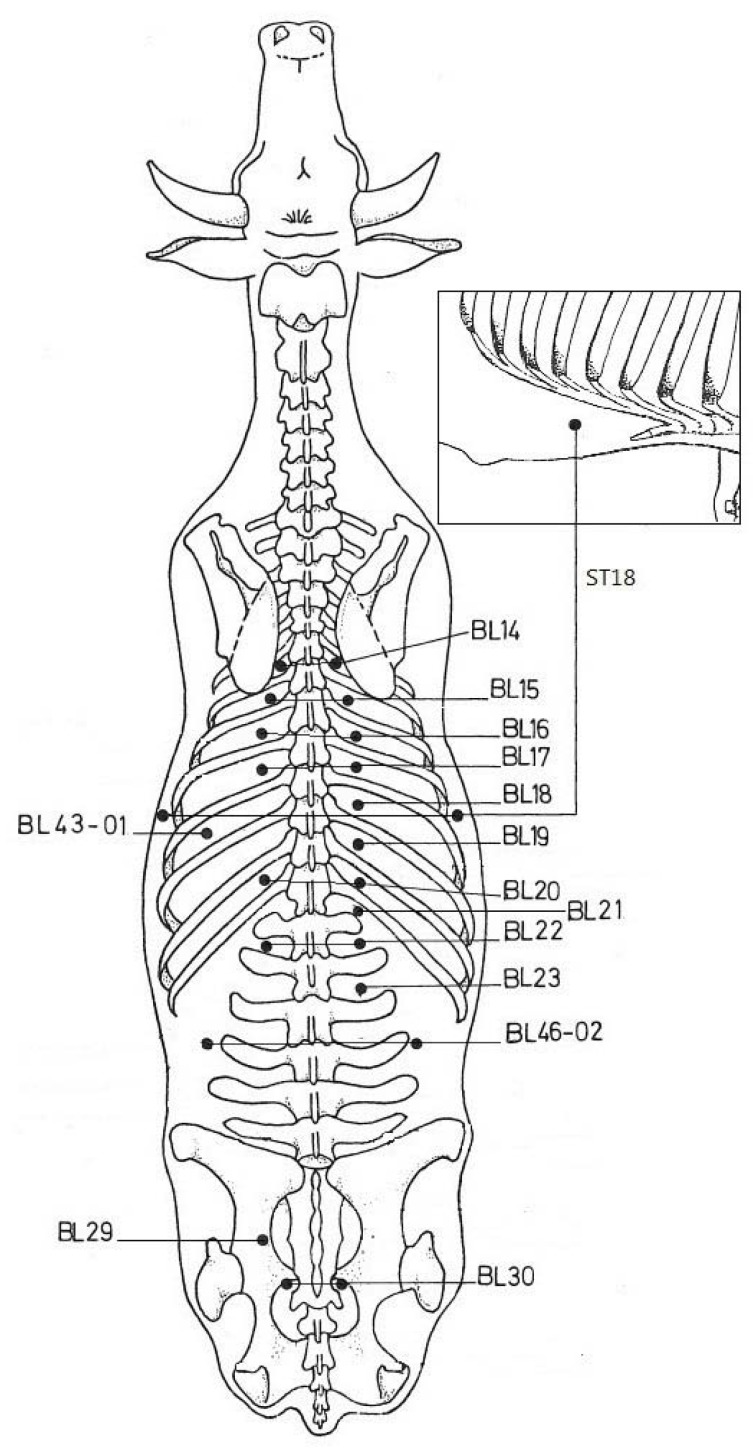
The location of the 24 measured APs (adapted from [8]).

The IV was measured with “Itronic” equipment from I-Health^®^ Systems [6]. The equipment used is a version of a device used to check the health of Soviet cosmonauts [7], and is used widely for human healthcare. For use in animals, the tip of the pen probe has been modified to be a trident instead of flat; this modification allows IV measurement without shaving (den Boer and Bosma, non-published data). Shaving may stress the cow and thus affect the IVs. If many APs must be measured, it is also time-consuming. The passive electrode of the measuring device was attached at the proximal end of a foreleg after applying some drops of water-based gel (AB-gel). The device’s software was designed to measure and register the impedance of 24 APs consecutively; *i.e.*, the 12 Ting points on the fingers and the 12 on the toes. We therefore selected 24 points for the experiments, choosing APs that tend to be clean and can be reached without putting instruments and health of practitioners at risk (Figure 1 and Table 1). We excluded APs at the distal limbs, and on the belly of the cow. The selection of the APs was based on information in [1] and [8,9,10,11].

**Table 1:** The sequence of measurement and the 24 APs, with codes according to IVAS [8].

Right sequence	1	2	3	4	5	6	7	8	9	10	11	12	13
	BL21	BL20	BL19	BL18	BL17	BL16	BL15	BL14	BL22	BL23	BL46-02	ST 18	BL30
Left sequence			14	15	16	17	18	19	20	21	22	23	24
			BL20	BL43-01	BL17	BL16	BL15	BL14	BL22	BL46-02	ST 18	BL30	BL29

IVAS = International Veterinary Acupuncture Society.

The IV measurements for Experiment 1 were performed in the Wageningen University herd (herd A), near Wageningen. For Experiment 2, cows were selected from three other Wageningen University herds: two conventionally managed herds (a low-tech herd (herd B) and a high-tech (herd D)), and an organically managed herd (herd C). Two herds used a milk robot.

### 2.1. Experimental Design

Experiment 1 (within-day and between-day effect) was carried out in a Latin square with six cows examined over three days and three measuring times per day (8:30–9:30 AM; 10:00–11:00 AM; 11:30–12:30 AM). Including a replication, this resulted in 108 measurements for each of the 24 APs. For Experiment 2, IVs were measured on the first three working days of three consecutive months: June, July and August. In each herd, 27 to 29 cows were selected from the available lactating cows. After three dates of measurement, 19 cows had been measured twice and 89 three times; thus in total, data on 108 cows were used for the analysis. As well as conducting IV measurements at the APs, the team observed and scored the body condition of the cows measured. The following information was retrieved from the records of the cows studied: breed, carrying fistula for digestion research (yes/no), year of birth, days in lactation, parity, number of inseminations, duration of previous lactation, kg milk, fat and protein in previous lactation, and: kg milk, % fat, % protein, % urea and cell count in milk from the current month. At the time of data-analysis, information for June and July was complete and those months were considered for the explanatory models (see Section 2.2). 

Before starting the experimental measurements, the team carried out AP localization and IV measurement four times (three times in herd A and once in herd B), to learn the routine. One person assisted and noted the body condition score and other observations of the cow, a second person held the laptop and monitored whether or not the measurement was successful; the Itronic does not register IV measurements outside the expected range (too high or too low), and requests a new measurement for that point. The third person (always the same one) located the APs and carried out the IV measurements.

### 2.2. Statistical Analysis

The statistical analysis was done with SAS software [11]. IV was measured in kΩ. The statistical analyses were done on log-transformed (ln) IVs. 

For Experiment 1, the time effect was verified with a linear regression (PROC REG) on the model: Y_ijkl_ = µ + day_i_ + cow_j_ + time_k_ + ε_ijkl_. If the distribution was normal for skewness, kurtosis and Shapiro-Wilk, then the within-day (*i.e.*, between hours), between-day and cow effects were tested with an ANOVA (PROC GLM), followed by the *post-hoc* Shapiro-Wilk test. If IVs were not normally distributed, a Wilcoxon test (PROC NPAR1WAY) was used instead, followed by the Kolmogorov-Smirnov *post-hoc* test. If there was no within-day effect, then for further analysis, the means of all six values of a day were averaged and used, together with the regression coefficient. To verify that the regression coefficients did not equal zero, we performed a two-tailed T-test (PROC TTEST). The correlation between APs was calculated according to Pearson (PROC CORR).

For Experiment 2, log-transformed means of the IVs per cow measured in one session were used to test the farm effect on the model Y_ij_ = µ + farm_i_ + ε_ij_. The log-transformed IVs were tested on normality before applying either an ANOVA or Wilcoxon test as above. The effect of days in lactation was tested on the model Y_ij_ = µ + lactation_i_ + ε_ij_. The lactation stage was averaged per cow over the 3-month period and then classified in five groups: 0–90; 91–181; 182–272; 273–363 and >363 attributed days. Depending on normality, either ANOVA (with Shapiro-Wilk *post-hoc*) or the Wilcoxon test (with Kolmogorov-Smirnov *post-hoc*) was used. An adjusted R^2^ regression followed by a stepwise procedure (*p* = 0.15), according to Hosmer *et al. *[12], was used to identify the model of best fit and the significant explanatory variables. 

## 3. Results and Discussion

### 3.1. Within-Day and Between-Day Effects on the Impedance Values at Acupuncture Points

The standard error of the means (SEMs) of the IV measurements at the individual APs were high (Table 2). The mean impedance and the SEMs on the left side were non-significantly higher than those on the right side: 1,965 ± 1,326 *versus* 1,761 ± 1,262 kΩ, respectively. However, this difference was less for the nine APs considered on both sides: 1,975 ± 932 compared to 1,836 ± 754 kΩ. The AP with the highest mean IVs was ST18 (3,618 ± 404 and 4,179 ± 418 kΩ); APs with the lowest IVs were BL30R (868 ± 59 kΩ), BL30L (827 ± 39 kΩ) and BL29L (856 ± 54 kΩ). The variation was greater between APs than within APs.

**Table 2:** Mean, median, standard error of the mean (SEM), highest and lowest impedance values in Experiment 1.

	Impedance values (kΩ)				
AP	Mean	Median	SEM	Lowest	Highest
BL21R	1,845	1,299	234.4	416	17,586
BL20R	1,971	1,389	215.7	357	13,450
BL19R	1,651	1,365	145.1	324	10,688
BL18R	1,318	1,143	102.9	419	10,102
BL17R	1,387	1,115	113.1	519	8,920
BL16R	1,710	1,133	173.6	269	12,042
BL15R	1,613	1,376	162.5	436	17,687
BL14R	1,757	1,281	153.4	57	9,674
BL22R	2,070	1,516	213.8	107	13,539
BL23R	1,554	1,084	197.6	107	13,555
BL46-02R	1,533	1,141	185.9	136	18,467
ST18L	3,618	1,833	403.6	584	18,358
BL30R	868	731	59.1	76	3,713
*R overall*	1,761	1,262		293	12,906
BL20L	2,495	1,548	331.5	292	18,267
BL43-01L	2,973	1,843	323.9	303	16,517
BL17L	1,899	1,359	215.6	259	14,829
BL16L	1,737	1,155	224.0	141	16,381
BL15L	1,605	1,300	164.9	155	16,800
BL14L	1,498	1,197	106.8	190	6,765
BL22L	1,775	1,285	186.3	297	16,431
BL46-02L	1,765	1,488	159.3	303	13,245
ST18L	4,179	1,918	417.8	589	17,814
BL30L	827	753	38.7	230	3,280
BL29L	856	739	53.8	235	3,485
*L overall*	1,965	1,326		272	13,075
*Overall*	1,854	1,291		283	12,984

**Table 3:** Results of the ANOVA (P) for the log-transformed impedance values (in kΩ) for the cow, within-day (Hr), and between-day effects.

		Variables		
AP	Mean ± SEM	Cow	Hr	Days
BL21R	7.24 ± 0.073	<0.01	-	-
BL20R	7.29 ± 0.070	-	-	-
BL19R	7.23 ± 0.072	<0.05	-	-
BL18R	7.05 ± 0.056	<0.05	<0.1	-
BL17R*	7.08 ± 0.083	-	-	-
BL16R	7.17 ± 0.105	<0.05	-	-
BL15R	7.23 ± 0.063	<0.1	-	-
BL14R	7.22 ± 0.104	<0.001	<0.01	<0.1
BL22R	7.36 ± 0.095	<0.001	<0.01	<0.05
BL23R	7.02 ± 0.119	<0.01	<0.1	-
BL46-02R	7.07 ± 0.095	<0.001	<0.01	<0.05
ST18R	7.75 ± 0.116	-	<0.1	-
BL30R	6.57 ± 0.101	<0.01	<0.1	-
BL20L*	7.44 ± 0.099	<0.05	-	-
BL43-01L	7.65 ± 0.072	<0.1	<0.05	<0.05
BL17L	7.27 ± 0.066	<0.1	-	-
BL16L	7.12 ± 0.104	<0.05	-	-
BL15L	7.18 ± 0.076	<0.05	-	-
BL14L	7.13 ± 0.099	<0.001	-	-
BL22L	7.24 ± 0.093	<0.01	<0.1	-
BL46-02L	7.31 ± 0.050	-	-	-
ST18L	7.93 ± 0.084	<0.05	<0.05	-
BL30L	6.62 ± 0.066	<0.001	<0.05	<0.01
BL29L	6.60 ± 0.094	<0.01	-	<0.1

* due to non-normality, the Wilcoxon test was performed

The cow effect was significant for most APs, except for BL 20R, 17R, 15R, 43-01L, 17L, 46-02L, and ST18R (Table 3). The within-day effect (hour) was significant for BL 14R, 22R and 46-02R (*p* < 0.01), and 43-01L, 30L and ST18R (*p* < 0.05). The between-day effect was significant for BL 22R, 46-02R, 43-01L (*p* < 0.05) and 30L (*p* < 0.01).

The mean regression coefficients showed a cow effect for BL17L, a within-day (*i.e.*, between-hour) effect for BL21R, and a between-day effect for BL14R. For BL22R the regression coefficient differed from 0. 

The log-transformed IVs at 22 APs were distributed normally, except for BL17R and BL20L (Table 3). Of the nine APs measured bilaterally, the IVs at five points (BL 14, 16, 17, 22 and 30) were significantly correlated (*p* < 0.001, *p* < 0.001, *p* < 0.05, *p* < 0.001 and *p* < 0.001, respectively), but the IVs at 4 APs (BL15, BL20, BL46-02 and ST18) did not correlate significantly with their pair. The IVs at BL14R, BL16R, BL16L and BL29L were significantly correlated with those measured at 20, 21, 19 and 20 APs respectively. The IVs at BL20L correlated more often with the IVs of APs located on the left, while those of ST18L correlated more often with the IVs of APs located on the right. The IV of BL43-01L did not correlate positively with the IVs of any other AP, but did correlate negatively with BL18R and BL19R (*p* < 0.05). IVs at BL20R, BL19R and BL46-02 correlated with IVs at only four other APs.

### 3.2. Effect of Dairy Production Factors on the Impedance Values at 24 Acupuncture Points

The mean IV on the left side was not significantly different than that on the right side; the overall mean and SEM were 1,993 ± 1,463 kΩ (Table 4). ST18 had the highest means (right: 3,427 ± 1,860 and left: 3,804 ± 1,876 kΩ). IVs were lowest at BL29L (1,269 ± 1,005 kΩ), BL30R (1,204 ± 1,014 kΩ) and BL30L (1,315 ± 1,860 kΩ).

Only ST18 did not show a farm effect. The IVs on all other APs were lower on farm B (conventional, no milk robot) than on D (conventional, milk robot). Most IVs from farm D were equal to those of farm C (organic, milk robot), except for BL 15R (*p* < 0.05), 17R (*p* < 0.05), 30R (*p* < 0.05), 14L (*p* < 0.01) and 43-01L (*p* < 0.05). The mean IVs were higher on farms C and D than on farms A and B.

For all three months, the period of lactation had a significant effect on BL43-01L (*p* < 0.05); the effect on BL16R was close to significant (*p* < 0.1). Days in lactation contributed significantly only to the June model for BL21R and to the July model for ST18R and BL30R. Parity contributed significantly to the July model for BL21R (*p* < 0.05); the contributions of parity to the July model for BL15R and ST18R were close to significant (*p* < 0.1), as were those for the June model of BL20R and BL18R (*p* < 0.1). Age contributed significantly to the June model for ST18L (*p* < 0.05), and its contribution was close to significant for BL15R and BL20L (*p* < 0.1)

In the June model, the variable “milk robot” contributed highly significantly to the model for most APs, except for ST18R, ST18L and BL30L. In the July model, the contribution was less important but was significant for nine APs. Other variables that contributed significantly to the models were the number of inseminations, body condition score, length of the preceding lactation, kg milk and kg milk fat of the current and preceding month and of the preceding year, and the cell count and the urea content in milk.

**Table 4:** Mean, median, SEMs, highest and lowest impedance values in Experiment 2.

	Impedance values (kΩ)				
AP	Mean	Median	SEM	Lowest	Highest
BL21R	1,940	1,498	132.4	319	16,716
BL20R	2,087	1,534	145.0	189	17,426
BL19R	2,098	1,549	141.3	162	17,448
BL18R	1,734	1,506	93.8	160	16,365
BL17R	1,799	1,446	127.1	216	18,427
BL16R	1,610	1,414	85.2	201	15,123
BL15R	1,675	1,417	87.5	319	15,365
BL14R	1,698	1,396	99.6	245	16,086
BL22R	1,930	1,468	142.5	170	18,211
BL23R	1,867	1,441	128.6	199	18,140
BL46-02R	2,238	1,523	162.1	324	17,858
ST18R	3,427	1,860	209.7	490	18,217
BL30R	1,204	1,014	77.4	232	16,451
*R overall*	1,939	1,467		248	17,064
BL20L	2,145	1,618	139.6	294	17,505
BL43-01L	2,720	1,683	186.4	326	18,264
BL17L	1,741	1,509	91.3	226	13,559
BL16L	1,854	1,469	119.2	133	17,467
BL15L	1,779	1,444	110.7	172	17,457
BL14L	1,928	1,403	137.9	132	16,622
BL22L	1,682	1,416	93.0	233	15,318
BL46-02L	2,493	1,541	180.0	363	16,907
ST18L	3,804	1,876	234.2	468	18,118
BL30L	1,315	1,081	82.9	200	15,468
BL29L	1,269	1,005	92.0	190	13,598
*L overall*	2,057	1,459		248	16,389
*Overall*	1,993	1,463		248	16,755

The adjusted R^2^ values were strongest for the models of BL14L in June and BL30R in July: 0.801 and 0.896, including 11 and 16 variables, respectively. The models of both ST18 left and right gave low R^2^ values in June but high in July. The R^2^ values of the models for right BL17, 18 and 30, and left BL 14, 17, 20, 29 and 43-01 were >0.5 in both June and July, including between 5 and 16 variables. For the stepwise regression, only the model for BL20L had a consistently high R^2^: 0.76 for June and 0.62 for July. The factor “milk-robot” dominated the model for BL30R in June but not in July. R^2^ values consistently >0.4 were scored by the models of right BL18, 19, 23, 29 and 30, and left BL 20 and 30. Overall, BL18R, BL30R, and BL20L gave consistently good correlations with the variables implemented, but the number of variables included was only 2 to 7. 

The variable most represented in the regression models was “milk robot”. For the month of June, its contribution was highly significant for 15 APs, and in July, it was significant for 8 APs. The IV of most APs was higher for cows from the two farms using a milk robot. This indicates either chronic health or nutrition problems. Other variables that contributed to the models and were also related to suboptimal health were the number of inseminations, the body condition score, the cell count and the urea content in milk.

### 3.3. Acupuncture Points Recommended for Future Use and Study

The within-day (between hours) effect for BL 14R, 22R, 46-02R, 43-01L, 30L and ST18L excludes these APs for standard EAP diagnostic procedures. This conclusion is reinforced by the effect of lactation period on BL43-01L, and of age on ST18L. The effect of days in lactation on BL21R and BL30R makes these APs less relevant; the contribution of parity to BL21R reinforces this conclusion. The near-significant (*p* = 0.1) contribution of parity to the monthly models of the effect of hour for BL18R, BL15R and ST18R, plus the near-significant contribution of age to the June model of BL15R and the near-significant effect of hour on BL18R and ST18R probably excludes these for diagnostic use. ST18 is related to the udder [8], which might explain the strong relation with age, parity and hour (Table 5). 

**Table 5:** The organs associated to the 24 APs according to Kothbauer [8].

APs	Associated organs right	Associated organs left
BL14	Pericardium	Pericardium
BL15	Heart	Heart
BL16	Lung	Lung
BL17	Lung, udder (right front)	Lung, udder (left front)
BL18	Liver, abomasum, omasum	-
BL19	Diaphragm, liver	Diaphragm
BL20	Spleen, pancreas	Spleen, pancreas
BL21	Ovary, indigestion	
BL22	Ovary, endocrine	Ovary, endocrine
BL23	Kidney, ovary, testis	-
BL29	-	Bladder
BL30	Udder (right back)	Udder (left back)
BL43-01	Rumen	Rumen
BL46-02	Small intestine	Large intestine
ST18	Udder (milk yield)	Udder (milk yield)

The non-normal distributions of IVs on BL17R and BL20L might be related to a physiological problem that we could not establish; they are recommended for further study, together with other APs for which no negative or positive advice can be given (Table 6). The differences between the IVs for BL17R and BL14L of cows from the conventional and organic farms that both use a milk robot argue for using these APs for diagnostics, but the first needs further study.

**Table 6:** The APs recommended for reference, diagnostics and further study.

Recommended for:	APs on the right					APs on the center			
Reference			BL19R	BL20R					BL46-02L
Diagnostics						BL14L	BL16L	BL17L	
Further study	BL16R	BL17R	BL18R	BL23R	BL30R	BL15L	BL20L	BL22L	BL29L

The non-significant cow effect for BL20R and BL46-02L and the correlation of BL19R with only four other APs, make these APs good reference points; BL46-02L is a suitable reference because of its low correlation with other APs. Disturbance at BL46-02 indicates intestinal disorders such as: diarrhea, catarrhal or regular enteritis, hemorrhage, obstipation, intestinal colic. Traditionally, the left side is more related to the colon, while the right side seems to be more related to the small intestine (Table 5).

Of the three APs shown in Table 6 as suitable for diagnostics, BL14L was chosen due to the high adjusted R^2^ value of the June model and BL 17L, due to the significant cow effect. BL16L has been added due to its correlation with 19 other APs. BL20L merits further study due to the high significance of two explanatory models and its non-normal distribution. Disturbances in BL14 and BL17 indicate respiratory and lung disorders; disturbances in BL14 also indicate functional heart disorders (Table 5). Disturbances in BL19R and BL20 may be indicative of liver disorders, upsets of abomasum or omasum, adrenaline deficiency, general weakness, and recovery after chronic disease, anemia and blood disorders; BL19 is also a pain point for peritonitis or pericarditis [8]. The use of BL45 (between the 11th and 12th rib), showing high IVs in herds with health stress [1], might also be considered.

We recommend pursuing the identification of normal values, effect of time (season), and of the most appropriate APs related to dairy cow performance, and to do so while developing and testing diagnostic and treatment equipment for use while the cow is standing in a milk parlor. It would also be worthwhile to investigate other as yet untested APs on the spleen and liver meridians. Hardware- and software-supported tools are now available that measure the reduction in the variation of the IV in one AP (ΔΚΩ/s) while a treatment is being applied [6]. This new software should be used on the APs indicated for diagnostics, to identify the optimal frequency of these waves for therapeutics. 

## 4. Conclusions

Hour of measurement significantly affected the IVs at six of the 24 APs measured; period of lactation affected three APs, and either parity or age affected a further three APs. The APs, BL19R, BL20R and BL46-02L had stable IVs and are suggested for reference measurements, and the BL APs 14L, 16L and 17L are recommended for diagnostics. The APs that merit further study are BL 16R, 17R, 18R, 23R, 30R, 15L, 20L, 22L and 29L. It would also be worthwhile to investigate other as yet untested APs on the spleen and liver meridians.

Variables that contributed significantly to the variation in the IVs of several APs were the use of a milk robot, the number of inseminations, body condition score, duration of the preceding lactation, kg milk and kg milk fat of the present and preceding month and of the preceding year, and the cell count and urea content in milk. We recommend pursuing the identification of normal values and seasonal effects, and the identification of the APs significantly related to dairy cow performance. The optimal frequency of sound, light or magnetic waves for specific therapeutics can be identified by means of new software that measures the reduction of variation in the IV when a frequency is applied to an AP.
